# Editorial: Altered metabolic traits in gastro-intestinal tract cancers, volume II

**DOI:** 10.3389/fendo.2026.1817958

**Published:** 2026-03-18

**Authors:** Seema Parte, Ranjith Kumavath, Ramesh Pothuraju, Saurabh Sharma, Rakesh Bhatia

**Affiliations:** 1 Department of Ophthalmology, Stanford University School of Medicine, Stanford University, Palo Alto, CA, United States; 2 Department of Biotechnology, School of Life Sciences, Pondicherry University, Puducherry, India; 3 Cancer Research Program, Rajiv Gandhi Centre for Biotechnology (RGCB), Thiruvananthapuram, Kerala, India; 4 Division of Surgical Oncology, Surgery Department, Stanford University School of Medicine, Stanford University, Palo Alto, CA, United States; 5 Amity School of Biological Sciences, Amity University Punjab, Mohali, Punjab, India

**Keywords:** colorectal cancer, gastro-intestinal tract cancers, lipid metabolism, liver metastasis, pancreatic cancer, serum metabolomics, triglyceride glucose index, tumor metabolism

## Introduction

**Figure 1:**
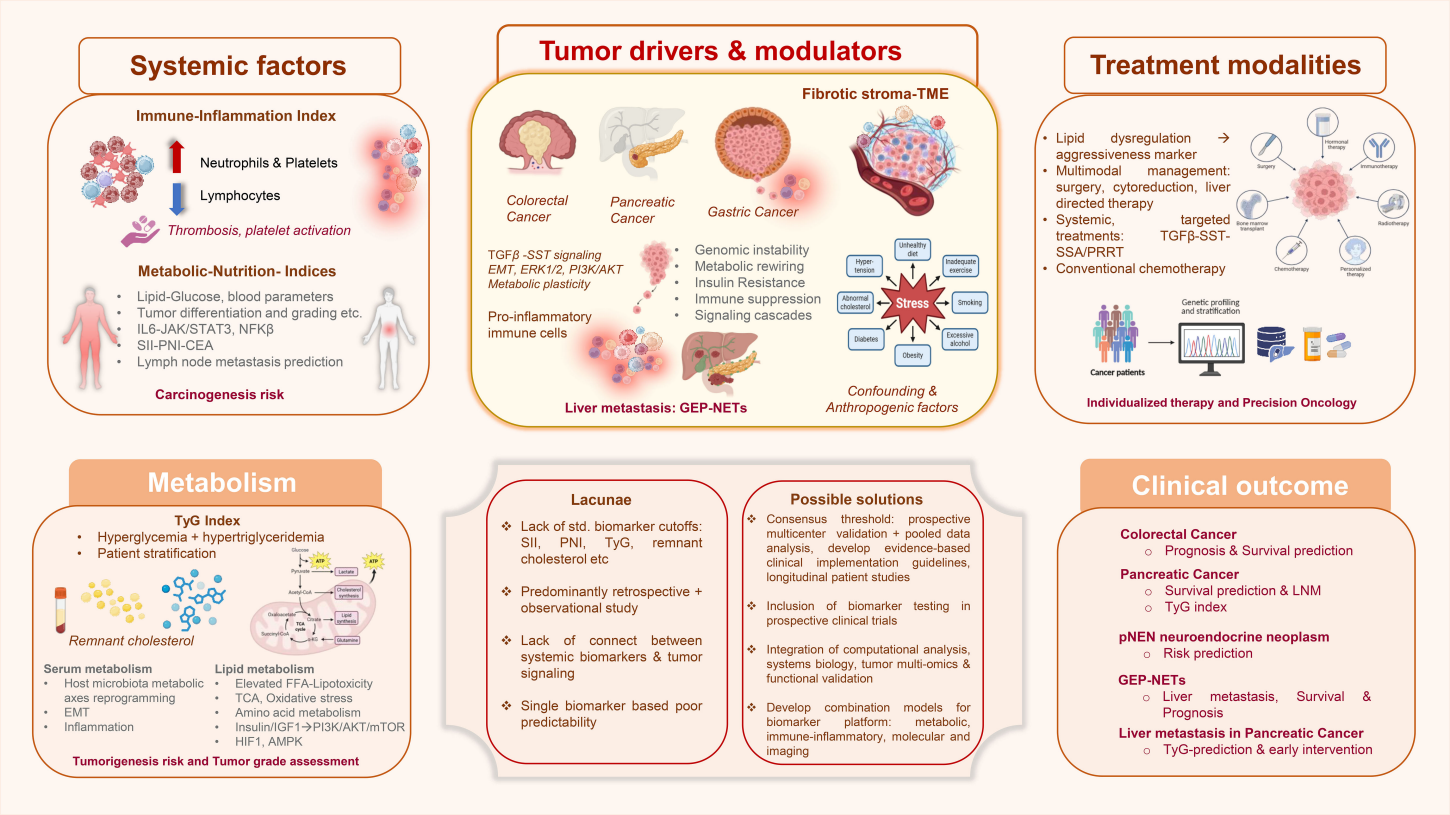
Schematic summary of gastric cancer early detection, prognosis and treatment strategies towards improved GI tract cancer: The schematic illustrates the complex array of multiple aspects including systemic factors such as immune-inflammation indices, metabolic-nutritional indices etc. besides the fundamental mutational landscape that predominantly drive, modulate and govern tumorigenesis, define carcinogenesis risk and subsequently determine clinical outcome. Tumor drivers include variables like fibrotic stroma dynamics from TME, metabolic rewiring, insulin resistance, immune suppression and signaling cascades besides genomic instability, involved in pan-GI cancer development, progression and metastasis. Specifically metabolic rewiring featuring TyG index, remnant cholesterol and serum metabolome stand out as modulators for assessing tumorigenesis risk and tumor grade assessment. Although treatment modalities warrant serious reformations, presently serum metabolic parameters represent good candidates, and lipid dysregulation determines tumor aggressiveness thus emphasizing the need for precision oncology through multimodal management. The schematic summarizes the lacunae that persist currently such as lack of standardized biomarker cutoffs, poor connectivity between systemic markers and tumor signaling etc. Proposed solutions include multicenter validation, multi-omics integration via computational analysis, systems biology approach and development of combination prediction models. Thus, various tumor types signify specific clinical outcome highlighting the requirement for early diagnosis, biomarker development, precise patient stratification, survival prediction and metastasis management. CEA, carcinoembryonic antigen; EMT, Epithelial mesenchymal transition; FFA, Free fatty acid; GEP-NETs, Gastroentero-pancreatic neuroendocrine tumors; GI, Gastrointestinal; pNENs, pancreatic neuroendocrine neoplasms; PNI, prognostic nutritional index; PRRT, peptide receptor radionuclide therapy; SSA, Somatostatin analogues; SST, Somatostatin; SII, system immune inflammation; TCA, tricarboxylic acid; TyG, Triglyceride glucose. Partially created with BioRender.com. Created in BioRender. Parte, S. (2026) https://BioRender.com/gs0d8fg. This presentation was created partly in BioRender.

Emerging knowledge in cancer biology reflects upon a critical understanding that cancer development, progression and prognosis is not merely dictated by alterations in oncogenic or tumor suppressor genes, but also by host tumor microenvironment (TME). TME reshapes tumor trajectory from progression to therapy response ultimately impacting patient survival (1). Articles in the Research Topic, volume 2 of Frontiers in Endocrinology, provide a concise understanding of various tumor driven local and systemic parameters (altered inflammatory, nutritional and metabolic indices) as useful biomarkers of disease onset, progression and prognosis across colorectal, gastric and pancreatic malignancies. For instance, gastric cancer (GC) is the third leading cause of cancer-related deaths worldwide (2). Approximately 75% of the patients present advanced disease during diagnosis (3). Pancreatic ductal adenocarcinoma (PDAC) is another major tumor type comprising 95% of the pancreatic cancer cases worldwide (4). Colorectal cancer (CRC) is among the most prevalent cancers globally and continues to impose substantial burden (5, 6), while developing from benign polyps to adenomas to ultimately invasive carcinoma (7, 8). These deadly cancers urgently require strategically improved diagnostics and biomarkers to curtail disease burden (**Figure 1**).

Here, a systemic review and meta-analysis by Wang and Jiang, encompassing ~26000 patients from 35 studies highlighted the prognostic significance of system immune-inflammation index (SII) in CRC. Since elevated pre-treatment SII consistently predicts poor overall and progression-free survival; inflammatory TME, immune suppression and pro-thrombotic states mediate tumor progression and metastasis. Additionally, lymph node metastasis (LNM) (9) is one of the major reasons behind poor prognosis of GC and current LNM diagnostic modalities (10) offer limited reliability. Addressing this concern, Dai et al. established the combined use of carcinoembryonic antigen (CEA), SII, and prognostic nutritional index (PNI), differentiation status, and tumor diameter in improving the diagnostic accuracy for LNM in GC patients versus either index alone (11). Integration of inflammatory and nutritional indices highlights a broader concept of tumor progression being influenced by systemic immunity and metabolic reserves, further encouraging composite biomarkers over specific laboratory test parameters. Predominantly retrospective design with variable cut-offs indicates a translational gap further encouraging prospective, multicenter initiatives to facilitate standardization and obtain translationally meaningful data.

Interestingly, metabolic markers may serve as powerful yet context dependent determinants of tumorigenesis. Recent study emphasized dysregulated lipid and glucose metabolism at the core of CRC carcinogenesis risk, and highlighted triglyceride-glucose index, (substitute of insulin resistance often associated with obesity, type2 diabetes etc) showing strong association with CRC incidence (12). Based on a meta-analysis of nine robust pooled observational studies, Wang et al., proposed that individuals with higher TyG values possess greater likelihood of developing CRC. This finding aligned well with the long-lasting evidence mechanistically linking metabolic imbalance, chronic inflammation, and CRC tumorigenesis; and supporting the TyG index as a low-cost, accessible tool for identifying high-risk individuals and guiding early preventive strategies.

Metabolic effects can exert variable repercussions across tumor types, whereby Yi et al., in a retrospective cohort study (n=172) identified the TyG index as a novel, reverse prognostic biomarker for pancreatic cancer liver metastasis. This study proposed a specific metabolic shift, i.e. transition from an insulin-resistant state to a catabolic equilibrium marked by reduced insulin secretion and cachexia-driven depletion of glucose and lipids. This was potentially intensified by cytokine-mediated inhibition of hepatic triglyceride synthesis within the metastatic niche. The study established TyG index as an accessible marker reflecting a distinct metabolic phenotype in advanced pancreatic cancer, necessitating further validation and mechanistic investigations. Tumor-directed local therapies, and a sequenced arsenal of systemic options, enabling personalized, precision-based treatment to optimize survival and quality of life are essentially required while managing aggressive metastatic tumor spread. From the viewpoint of managing liver metastasis in gastroenteropancreatic neuroendocrine tumors, Xue et al., systematically reviewed this subject and reported that surgical resection and cytoreductive therapy offer most favorable survival outcome, whereas liver directed therapies like ablation and embolization could address unresectable cases. A systemic approach could manage symptoms and control disease progression; however, the requirement of prospective comparative studies cannot be negated.

Further, Zhou et al., explored the association between remnant cholesterol and tumor grade in pancreatic neuroendocrine neoplasms (pNENs) indicating dysregulated lipid metabolism with tumor aggressiveness. Lipids being critical components of cell membrane, signaling molecules, and bioenergetics; circulating lipids represent tumor metabolic reprogramming (13, 14). Findings suggest that remnant cholesterol is a useful biomarker for risk stratification and tumor grading, though further investigation into the underlying mechanisms is needed. Integrating multiomics-based diagnostic study in CRC may help to bridge the systemic associations with mechanistic insights, where altered lipid, amino acid and energy metabolism signatures could serve as non-invasive biomarkers. In this context, the study by Wang et al., provided mechanistic insights into CRC metabolism with emphasis on serum metabolomics, in combination with multiomics validation. This may serve as promising approach for early diagnosis, but nevertheless, large prospective clinical cohort studies are warranted.

Dysregulated MYC for instance, is implicated in therapy resistance of neuroendocrine tumors. However, tumor heterogeneity and therapeutic resistance due to dysregulated crosstalk between TGF-β and somatostatin signaling in pancreatic adenocarcinoma and neuroendocrine tumors were comprehensively reviewed by Ungefronen et al. TGF-β dictates epithelial to mesenchymal transition (EMT), immune modulation and tumor progression, whereas somatostatin pathways exert inhibitory and differentiating roles. TGF-β being pleiotropic, it exerts variable effect during tumor evolution and its crosstalk with other signaling hubs require further investigation. Collectively studies infer that gastro-intestinal (GI) cancers evolve as systemic metabolic diseases, encompassing immune-inflammatory activation, insulin resistance, lipid dysregulation thus orchestrating tumor-intrinsic signaling.

## Strategic foresight

Tumor evolution, its burden, aggressiveness, heterogeneity and therapeutic responsiveness are key determinants for the choice of tumor-directed therapies (either combined/targeted), enabling optimal survival and quality of life. Computational analysis and systems biology based- comprehensive, integrated approach combining multiomics, metabolomics, immune and molecular signatures may offer novel avenues for risk assessment, biomarker development and enabling personalized oncotherapeutic strategies with greater precision for holistic management of GI cancers. Together these studies could positively impact drug designing and efficient patient stratification, survival prediction and metastasis management based on above mentioned considerations with promising outcomes.
